# African swine fever virus: A re-emerging threat to the swine industry and food security in the Americas

**DOI:** 10.3389/fmicb.2022.1011891

**Published:** 2022-10-05

**Authors:** Julian Ruiz-Saenz, Andres Diaz, D. Katterine Bonilla-Aldana, Alfonso J. Rodríguez-Morales, Marlen Martinez-Gutierrez, Patricia V. Aguilar

**Affiliations:** ^1^Grupo de Investigación en Ciencias Animales—GRICA, Universidad Cooperativa de Colombia, Bucaramanga, Colombia; ^2^PIC—Pig Improvement Company, Querétaro, Mexico; ^3^Grupo de Investigación Biomedicina, Faculty of Medicine, Fundación Universitaria Autónoma de las Américas, Pereira, Colombia; ^4^Faculty of Health Sciences, Universidad Cientifica del Sur, Lima, Peru; ^5^Grupo de Investigación en Microbiología Veterinaria, Escuela de Microbiología, Universidad de Antioquia, Medellín, Colombia; ^6^Department of Pathology, University of Texas Medical Branch, Galveston, TX, United States; ^7^Center for Tropical Diseases, Institute for Human Infection and Immunity, University of Texas Medical Branch, Galveston, TX, United States

**Keywords:** African swine fever, reservoirs, Arbovirus, emerging disease, pigs

## Introduction

African swine fever (ASF) is a devastating disease for the swine industry, characterized by hemorrhagic fever with up to 100% mortality rate, and with a tremendous socioeconomic impact worldwide (Dixon et al., 2020). The disease was first reported in East Africa in the early 1920s as an acute hemorrhagic fever that caused the death of almost all infected domestic pigs (Montgomery, 1921; Plowright et al., 1969). Since then, the African swine fever virus (ASFV) has remained endemic in Africa affecting up to 35 African countries and has emerged in Europe, Asia, and now in the Americas.

## Reemergence of ASFV

Since the reemergence of ASFV in Europe through the Caucasus region in 2007, the virus has rapidly expanded and reached the Russian Federation, East Europe, and Asia (Dixon et al., 2020). In 2018, the detection of ASFV in China, killing at least half of the swine population of China (Zhou et al., 2018), and subsequent dissemination to Southeast Asia; threat one of the most significant swine industries of the world, which contains half the world's swine population (Dixon et al., 2020; Gaudreault et al., 2020). The high socioeconomic impact of ASFV infection results from direct death and culling of the animals and loss of business in the swine production chain, costs of disease control, and international trade blocks (Zhou et al., 2018). In addition, large epidemics can result in dramatic reductions in the size of national pig herds and inflation of prices of pig and pork products (Dixon et al., 2020; Gaudreault et al., 2020).

It is well-known that the highly virulent ASFV genotype II that emerged in the Caucasus region in 2007 is responsible for the contemporary European/Asiatic epidemic (Sánchez-Vizcaíno et al., 2013; Ge et al., 2018; Cwynar et al., 2019). The virus has spread to domestic pigs and wild boards across Eastern Europe and Asia (Gavier-Widén et al., 2015; Li et al., 2019). Multiple efforts are in place to avoid the dissemination of the ASFV throughout the European Union. However, the disease was first identified in Lithuania, Poland, Latvia, and Estonia in 2014 (de la Torre et al., 2015), and by 2019, it was in Belgium, Bulgaria, Slovakia, Estonia, Hungary, Latvia, Lithuania, Poland, and Romania. By the end of 2020, Germany reported their first case of ASF in domestic pigs (Sauter-Louis et al., 2021b). The current epidemiology of ASF in wild boars in East Europe plays a vital role in the risk of ASFV transmission to the domestic population (Sauter-Louis et al., 2021a; de la Torre et al., 2022).

In the Americas, ASFV emerged in late 1970s in Brazil, Cuba, and the Caribbean Island with a full eradication at early 1980s (De Paula Lyra et al., 1986). Nevertheless, ASFV re-emerged in the Americas in 2021. On 28 July 2021, the United States Department of Agriculture (USDA, 2021) confirmed the presence of ASFV in the Dominican Republic and it turned on the alarms for the swine industry in the Americas. According to the Department of Agriculture of the Dominican Republic, the virus has been detected in at least 22 out of the 31 provinces in the country (Agricultura, 2021). As stated by the World Organization of Animal Health (WOAH), the genotype II was detected in all positive samples (WOAH, 2021a,c).

On 20 September 2021, the Chief Veterinary Officer from Haiti reported a new case of ASF to the WOAH, becoming the second Country with ASFV positive samples in the Americas. The sample was collected from a backyard farm in a province bordering the Dominican Republic and was tested by the USDA Laboratories through a cooperative testing program. The report filed with the WOAH indicates the outbreak in Haiti began in late August and killed several swine (WOAH, 2021d) and by the end of October 2021, a total of seven outbreaks of ASF have been identified, affecting multiple provinces all over the country (WOAH, 2021b).

With a case fatality rate close to 87% in the Dominican Republic (WOAH, 2021a), lack of approved vaccines, the current ASF outbreaks highlight the devastating consequences for the swine industries (Busch et al., 2021). ASFV is now confirmed in at least 60 countries worldwide (in which ~80% of the swine population resides). Furthermore, global ASF outbreaks have increased 25% since 2018 (Gaudreault et al., 2020), changing the swine industry's global dynamics.

ASFV is a complex, enveloped virus that contains a large (170–190-kb) double-stranded DNA (dsDNA) genome (Tulman et al., 2009). ASFV is the only known member of the *Asfarviridae* family (Alonso et al., 2018) and it is currently grouped into 24 genotypes, all of them being associated with disease (Achenbach et al., 2017). Although most of the genotypes have been linked to ASF outbreaks in various parts of sub-Saharan Africa, genotype I dominates in Central and West Africa (Minoungou et al., 2021; Njau et al., 2021). The first description of AFSV outside Africa, was reported in 1958 and again in 1961 from Lisbon Portugal with subsequent spread to other regions of Europe and Latin America of the genotype I (Mur et al., 2016). During the 1970s and 1980s, ASF become endemic in the Iberian Peninsula and many other countries were affected by sporadic ASFV outbreaks due to the introduction and use of food waste from international planes or boats to feed pigs. Strong and long-lasting efforts were put in different places to achieve eradication in most of Europe, for a comprehensive review see Danzetta et al. (2020).

Virulence is not fully associated with the viral genotype, and infection can lead to a broad spectrum of disease severity, from highly lethal to subclinical or asymptomatic, depending on host characteristics and the specific viral strain (Boinas et al., 2004). While a highly virulent ASFV has been widely reported in Eurasia (90–100% mortality in domestic pigs and European wild boar), reduced virulence strains and attenuated strains of the ASFV genotype II were found in Estonia and Latvia (Zani et al., 2018; Gallardo et al., 2019; Vilem et al., 2020) suggesting a threat for control programs due to the risk of pigs with chronic and carriers behaviors besides of the difficulty of early detection of ASF epizootics due to a lack of clear specific clinical signs of infection, as well as the presence of non-viremic animals (Gallardo et al., 2018).

Based on genotyping, whole genome sequencing and phylogenetic analysis, the ASFV that spread through Europe (Georgia 2007/1) has been proved to originate in South East Africa, but the exact location of the origin of this genotype includes Mozambique, Malawi, Zambia, southern Tanzania, and Madagascar (Quembo et al., 2018; Njau et al., 2021). However, the recently confirmation of the ASFV genotype II in Lagos, Nigeria (West Africa), complicates the already constrained control measures against the disease in the region (Adedeji et al., 2021) and emphasize the risk of worldwide dissemination of this highly pathogenic genotype II.

## Vectors and host/reservoirs

ASFV can also be transmitted by soft ticks of the genus *Ornithodoros* in the family *Argasidae*, which act as biological vectors of ASFV (Pereira De Oliveira et al., 2019). To date, eight *Ornithodoros* taxa have been demonstrated as competent vectors of ASFV, including *O. marocanus, O. puertoricensis, O. coriaceus, O. moubata porcinus, O. erraticus, O. moubata complex, O. turicata , and O. savignyi* (Groocock et al., 1980; Mellor and Wilkinson, 1985; Hess et al., 1987; Endris et al., 1991, 1992; Kleiboeker et al., 1998; Rennie et al., 2000; Ribeiro et al., 2015; Golnar et al., 2019). Although *O. erraticus* ticks has been proven competent to replicate the ASFV genotype II, it failed to transmit the Eurasian ASFV strains to naive pigs (Diaz et al., 2012; Pereira De Oliveira et al., 2019) under experimental conditions, suggesting that other determinants beyond viral replication also influence ASFV vector competence (Pereira De Oliveira et al., 2020b). However, recent analysis had shown successful infection of domestic pigs by ingesting *O. erraticus* ticks that fed on ASFV-infected pigs suggesting that *O. erraticus* may act as a reservoir of ASFV and suggesting new transmission routes of ASFV (Pereira De Oliveira et al., 2020a).

In the Americas, multiple *Ornithodoros* species could be considered potential vectors of ASFV. At least three *Ornithodoros* species found in the US are considered high-risk competent vectors (*O. coriaceus, O. turicata*, and *O. puertoricensis*). However, it remains unknown if other soft ticks in the Americas can also transmit ASFV (Golnar et al., 2019). Critical role of certain *Ornithodoros* species in the Americas and the Caribbean need to be clarified since *O. puertoricensis* could be an efficient vector for ASFV (Butler and Gibbs, 1984; Butler et al., 1985). Nevertheless, the presence of these ticks in Haiti and the Dominican Republic did not appear to complicate the eradication of AFV from these countries in 1978 possibly due to a lack of contact between infected pigs and *O. puertoricensis* (Kleiboeker and Scoles, 2001) or perhaps due to a low stadial transmission of ASFV in the *O. puertoricensis*, which was found to decrease from nearly 100% to less than 35% from the nymphal to adult stage (Endris et al., 1991) that reduced the risk of transmission. This low transmission could be due to the presence of endogenous viral elements of the ASFV, which might have been integrated into soft tick genomes and serve as templates for siRNA and piRNA thereby possibly protecting the tick against viral infection as has been recently reported for *O. moubata* and *O. porcinus* field-collected ticks from Africa (Forth et al., 2020).

In regard to competent hosts, at least three susceptible species of swine are present in the Americas: domestic pigs (*Sus scrofa domesticus*), the invasive feral boars (*Sus scrofa*) in wildlife, and common warthogs (*Phacochoerus africanus*) in different zoos (Golnar et al., 2019). ASFV is easily transmitted from persistently infected host/reservoirs to uninfected animals (de Carvalho Ferreira et al., 2013; Gallardo et al., 2015). Therefore, the possibility that wild fauna in the Americas can become persistently infected and spread the virus to susceptible animals urge to be considered (Brown and Bevins, 2018). In fact, a recent analysis of the risk of ASFV establishment and spillover in the United States has been reported. Based on feral swine distribution, soft ticks, and the inventory of domestic swine in the US this report indicates that certain areas of California, Florida, and much of the southwestern United States are high risk zones for ASFV establishment and spillover (Wormington et al., 2019).

Boar hunting and human movements across borders with contaminated fomites or meat represent the major risk for naive populations. Hence, the presence of the ASFV genotype II in the Dominican Republic and Haiti highlights the urgent need for better transnational discussion regarding but not limited to (1) preventive measurements; (2) appropriate disposal of swine products potentially contaminated with ASFV; (3) early detection of the virus if introduced into a naive country; (4) prompt and coordinated response of producers, local authorities, and governments; (5) education; and (6) communication between countries. This discussion should include not only governments and decision-makers, but also large, medium, and small-sized pork producers.

## Prevention, control, and preparedness

Despite prevention and control efforts, ASF has led to an unprecedented crisis in the global pig sector representing a global risk to animal health and welfare, national and international economies, rural development, social/political behavior, national food security, and national and international markets (Gf-TADs, 2020). The Global Framework for the Progressive Control of Transboundary Animal Diseases (GF-TADs), funded by WOAH, and FAO has established a plan for preparation and early detection of a possible arrival of ASFV to the Americas (Komal, 2020). Once the virus was reported in China, countries of the Americas came together to discuss prevention and to establish preparedness plans. However, this was not enough to avoid the arrival and dissemination of the ASFV in the Dominican Republic and the subsequent transborder transmission to Haiti. The economic impact of ASFV in the pork industry of the Americas could be devastating. Five out of 15 of the major pork exporters of the world are in the Americas and the presence of ASF in a country will limit its trade capabilities of pork.

**Figure 1:**
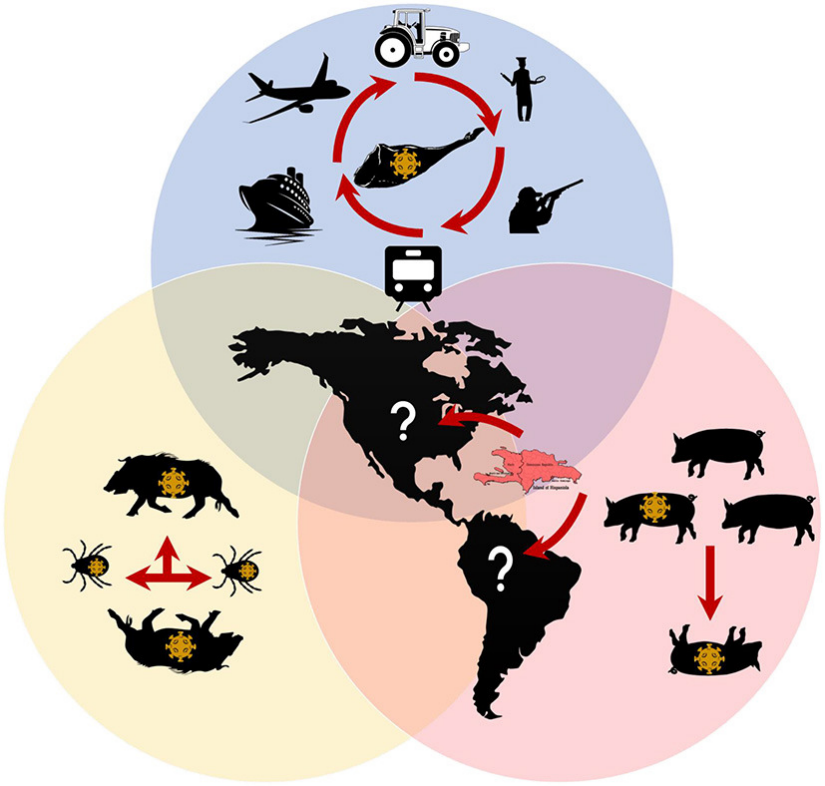
ASFV transmission known and gaps in the Americas. Red arrows represent potential routes for ASFV transmission. **Blue shape**: Human movement with contaminated fomites or contaminated pork represent the major risk for naive populations. **Yellow shape**: The potential role of wild Boars and *Ornithodoros* tick vectors still to be determined in the Americas. **Orange shape**: Virus transmission in pig farms has been confirmed in the Dominican Republic and Haiti. See text for references.

Given the current global situation and the detection of the virus in the Dominican Republic and Haiti, the swine industry recognizes a high risk of introducing ASFV into the continental part of the Americas (Figure 1). Hence, multiple preparedness efforts are being established in each country to avoid introducing and disseminating the disease. Responsible importation of supplies potentially contaminated with ASFV and appropriate downtime for hard-to-clean and disinfecting supplies is encouraged. Strict biosecurity practices and compliance at all swine production and management levels should be mandatory if producers want to keep the disease out of their system.

Since ASF is not a public health risk, people can regularly eat pork or pork-derived products. However, no pork or pork products should be transported from a country with ASF to another country. Additionally, no pork or pork products should be allowed into swine production facilities, given the risk of introduction through contaminated pork. ASFV DNA has been found in multiple pork products brought into South Korea and Taiwan by travelers (Kim et al., 2019; Wang et al., 2019), although no live virus has been isolated from such products. Furthermore, pigs should not be fed with food waste from restaurants or any kitchen. Clean and dirty areas must be clearly defined at feed mills, swine farms, and truck wash facilities (Li and Tian, 2018). There should be biosecurity protocols in place to avoid the contamination of feed or supplies for feed manufacturing. All supplies must be cleaned and disinfected before crossing into the clean area of swine facilities. Moreover, every swine production facility must have a response plan including management and disposal of mortality in case of an ASF outbreak.

Backyard production may possess different risks or perceptions regarding the disease than industrialized swine production. Hence, communication between authorities, producers, and local governments is key to reduce the risk of dissemination if the virus hits a given country. Active surveillance at different levels is required for early detection. Early detection of the virus is a crucial component minimize the impact of ASF introduction into a country. Unfortunately, in the Americas, PCR diagnosis can only be attempted until a suspect case is reported. Hence, if the clinical manifestation of the disease happens 2–3 weeks after the introduction into a farm, it might be too late to detect the virus (2–3 weeks after introduction into a commercial swine farm) and avoid dissemination within a region given the continuous flow and movement of pigs in the modern swine industry. Several European Union countries allow producers to monitor ASF status in the farms without needing to wait for clinical signs. It is also important to denote that recently based on expert perceived opinion, passive surveillance of wild boar and syndromic surveillance of pig mortality has been considered to be the most effective for controlling ASFV spread, whereas culling of all infected herds and movement bans for neighboring herds were considered as the most effective intervention strategies (Guinat et al., 2017).

As important measures, the USDA has recommended to the WOAH to establish the first foreign animal disease protection zone in United States, Puerto Rico, and the US Virgin Islands. Also, the Animal and Plant Health Inspection Service (APHIS) has suspended movement of live swine, swine germplasm, swine products, and swine byproducts from Puerto Rico and the US Virgin Island in an effort to keep ASF out of the US mainland. The establishment of an WOAH officially recognized protection zone would allow the US to maintain its ASF disease-free status, continue exporting pork and live swine, even if ASF is discovered within the protection zone (APHIS-USDA, 2021).

A new eradication of ASFV from the Haiti and the Dominican Republic can be achieved in spite we are facing a different epidemiological context. Previous eradication in the Americas showed that classical surveillance strategies, both at farm and slaughterhouse levels, conventional biosecurity and sanitary measures and well-structured collaboration among institutions in affected countries hand-in-hand to the international organizations (FAO and WOAH) is keystone for ASFV eradication (Danzetta et al., 2020). Countries should strengthen their surveillance systems based on previously known capabilities developed through the current “Continental Plan for the eradication of Classical Swine Fever (CSF) from the Americas,” that has achieved a CSF free status in most of the American continent in a step-by-step process of management, control, and elimination of the disease in infected countries (Ganges et al., 2020; WOAH, 2021e). The continuous support from USDA and other high-income countries will be crucial for the ASFV contention and eradication in the Hispaniola Island, which could be more economically profitable than assuming the losses of a possible arrival of the ASFV to continental soil.

The current economic crisis in Latin American countries like Venezuela (Suárez et al., 2018) highlights a possible risk of ASF introduction into South America, given the high probability of pork importation products to Venezuela from ASFV-positive countries. In addition, the current COVID-19 pandemic seems to be negative for the control of the ASFV. Low-income countries in Africa have reported increasing ASF incidence in association with the coronavirus emerging disease, mainly due to the redistribution of resources and attention toward mitigating the COVID-19 pandemic (Uwishema et al., 2021). It is essential to consider the socio-political conditions of the many nations of the Caribbean. The introduction of ASFV into Haiti and its dissemination throughout the country may increase the strong socio/economic disparities, and long-standing governance problems as well as ecological disturbance due to Natural disasters and uncontrolled COVID-19 infections (Oxford-Analytica, 2020; Hoffman, 2021). The same could apply for Cuba and other Caribbean nations in geographic proximity (Gorry, 2021); the close relationship, international trading and people movement between positive and naive nations could favor ASFV transmission.

## The urgent need for a vaccine

Finally, no effective ASFV vaccine exists commercially. However, multiple vaccine developments are in progress due to the current situation of ASF worldwide (Bosch-Camós et al., 2020), Vaccine efficacy and vaccination strategies is a subject that needs to be taken into consideration to prevent a possible dissemination of ASFV in the Americas (Rivera-Benítez et al., 2021). Multiple vaccine development strategies have been employed, with varying levels of success (Gaudreault et al., 2020). The most promissory vaccine candidate has been achieved by deletion of the ASFV I177L gene (ASFV-G-ΔI177L) resulting in sterile immunity against the epidemic ASFV Genotype II (Borca et al., 2020). Besides, this attenuated ASFV strain has proven being effective to protect pig breeds against genotype II ASFV challenge (Tran et al., 2021) and completely protected against virulent ASFV challenge when it was administered by the oronasal route, even at large-scale experiments (Borca et al., 2021b; Tran et al., 2022). The stable adaptation of this live attenuated vaccine strain to cell culture (Borca et al., 2021a) and its oronasal distribution will allow to produce an ASF vaccine on a commercial scale and it possible use for wildlife. Moreover, an accompanying genetic test to discriminate between infected and vaccinated animals (DIVA) has been developed, and are a promising option to support the control and eradication of the ASFV genotype II during a potential vaccination program (Velazquez-Salinas et al., 2021).

## Concluding remarks

Despite the multiple multilateral efforts to avoid ASFV emergence into the Americas, the virus has arrived and spread rapidly from one country to another. This situation highlights the need to establish local, regional, and transnational measures to avoid the spread of ASFV to other countries in the region (Kading et al., 2019). It is imperative to establish and follow national and international guidelines on preventive surveillance and diagnosis including enhancing communication among countries, strengthen vector surveillance, and increase public awareness of ASFV. Finally, we encourage to establish regional networks of cooperation, as has been implanted in the European Union (EFSA et al., 2021), that help to research/understand major gaps in knowledge in the Americas as much as to harmonize diagnostic techniques, share real-time surveillance data and help to avoid the possible dissemination of ASFV in the Region.

## Author contributions

All authors listed have made a substantial, direct, and intellectual contribution to the work and approved it for publication.

## Funding

This work was financially supported CONADI-UCC Grant to JR-S and by the Fogarty International Center and the National Institute of Allergy and Infectious Diseases, of the National Institutes of Health under Award Number D43 TW010331 to PA. The content is solely the responsibility of the authors and does not necessarily represent the official views of the National Institutes of Health.

## Conflict of interest

Author AD is employed by Pig Improvement Company. The remaining authors declare that the research was conducted in the absence of any commercial or financial relationships that could be construed as a potential conflict of interest.

## Publisher's note

All claims expressed in this article are solely those of the authors and do not necessarily represent those of their affiliated organizations, or those of the publisher, the editors and the reviewers. Any product that may be evaluated in this article, or claim that may be made by its manufacturer, is not guaranteed or endorsed by the publisher.
